# A Dual-Field Sensing Scheme for a Guidance System for the Blind

**DOI:** 10.3390/s16050667

**Published:** 2016-05-11

**Authors:** Qing Lin, Youngjoon Han

**Affiliations:** 1 Sports-IT Convergence Department, Soongsil University, 511, Sangdo-Dong, Dongjak-Gu, Seoul 156-743, Korea; linqing15@ssu.ac.kr; 2 Department of Smart Systems Software, Soongsil University, 511, Sangdo-Dong, Dongjak-Gu, Seoul 156-743, Korea

**Keywords:** electronic travel aids, guidance systems for blind people, sensor fusion, scene interpretation, probabilistic graphical model, context awareness

## Abstract

An electronic guidance system is very helpful in improving blind people’s perceptions in a local environment. In our previous work “Lin, Q.; Han, Y. A Context-Aware-Based Audio Guidance System for Blind People Using a Multimodal Profile Model. *Sensors **2014**, 14,* 18670–18700”, a context-aware guidance system using a combination of a laser scanner and a camera was proposed. By using a near-field graphical model, the proposed system could interpret a near-field scene in very high resolution. In this paper, our work is extended by adding a far-field graphical model. The integration of the near-field and the far-field models constitutes a dual-field sensing scheme. In the near-field range, reliable inference of the ground and object status is obtained by fusing range data and image data using the near-field graphical model. In the far-field range, which only the camera can cover, the far-field graphical model is proposed to interpret far-field image data based on appearance and spatial prototypes built using the near-field interpreted data. The dual-field sensing scheme provides a solution for the guidance systems to optimise their scene interpretation capability using simple sensor configurations. Experiments under various local conditions were conducted to show the efficiency of the proposed scheme in improving blind people’s perceptions in urban environments.

## 1. Introduction

Electronic travel aid (ETA) devices [1] are very helpful for blind people when they traverse a local environment. Compared to white canes, these ETA devices can monitor a wider area and enhance a blind user’s perceptions via various feedback modalities. In general, ETA devices can be categorised based on the sensors they use to monitor the environment and the feedback schemes they provide to the user. Sensors that are widely used in these devices include ultrasonic sensors [2,3,4,5], laser sensors [6,7,8,9], cameras [10,11,12,13,14,15,16,17] and Kinect sensors [18,19,20,21,22]. The common feedback modalities are either auditory or tactile.

A critical task for ETA systems is to interpret the current scene for the blind user. This interpretation capability can be measured in terms of the interpretation resolution and scale for the scene. A scene interpretation scale spans three dimensionalities of a physical scene. This scale is usually determined by the sensor configuration of an ETA system. In the forward dimension, a preview distance of 10–15 m would be sufficient for a guidance task. In the horizontal dimension, a viewing angle of 120° might give the blind user a good experience of a normal human view range. In the vertical dimension, the interpretation scale should cover from the head of a normal person down to the ground level. Because ETA devices are usually attached to human bodies, the sensors used to cover the three-dimensional (3D) scales should be as simple as possible.

Scene interpretation resolution can be divided into three levels. The low level is simply to identify free space. The medium level is to identify the location of object groups that can be represented in regular grids [13] or polar grids [16]. The high level is to identify the status of each single object, including its location, size and object type. There is no doubt that the high interpretation level could provide the blind user with a more advanced user experience. However, there are many challenges to realise the high interpretation level on an ETA platform. First, the portable design of an ETA platform allows only simple sensors with quite limited sensing properties. Second, the quality of sensor data could suffer from the shaky motion of the blind user. Third, the complexity of objects in a real scene makes it rather difficult to identify each object reliably. Due to the above challenges, most existing ETA systems can only achieve the medium interpretation level.

The scene interpretation capability of an ETA system should be sophisticated for a better user experience in a complicated environment. Therefore, we believe that the future development of ETA sensing systems should include distinct advancements in the following two aspects of scene interpretation capability: (1) cover sufficient interpretation scale with simple sensor configuration; (2) increase interpretation resolution in each scale dimension.

To achieve these goals, we propose an ETA sensing system that only uses a two-dimensional (2D) laser scanner and a camera that are fixed on a human waist [23]. In the proposed system, several strategies were designed to improve the scene interpretation capability. First, the 2D laser scanner and camera is fixed at a downward-pointing angle to cover ground-level objects in addition to high-level objects. Laser range data are used as the main cues for estimating the locations of the ground and of each object. Second, to interpret each object’s type, the camera is used as a complement to the laser scanner. By integrating range profiles from the laser and edge profiles from the image, a multimodal profile model of object is built. Using this model, generic objects on the road can be classified into seven categories. Third, in order to handle uncertainties in scene interpretation, the ground and each object’s state are jointly inferred using a graphical model that produces the final interpretation results.

Following the above strategies, high interpretation resolution can be achieved in each scale dimension. However, a downward-pointing laser scanner can only cover a forward range up to five metres. Therefore, the near-field graphical model that requires data from both laser and camera can only be applied in this near-field range. This limited interpretation scale in the forward dimension can cause trouble in searching for the best walking direction and may even lead to dead ends during guidance. Moreover, limited knowledge in the forward dimension also makes it difficult to predict the overall walking context [23] properly, which may result in some misleading guidance behaviours.

To solve these problems, a far-field graphical model is proposed in this paper to extend the interpretation scale in the forward dimension. It takes multiple clues from the output of the near-field model to interpret the far-field image data adaptively. Finally, by integrating interpretation results from the near-field and far-field model, a local map with richer information about the scene can be generated. Based on this map, more intelligent guiding service can be provided to blind people.

Although the far-field model extends the interpretation scale in the forward dimension, it also adds extra computational load to the system. To ensure real-time performance, the near-field graphical model is also improved. A scout point-voting scheme is developed to boost object profile clustering so that object detection in the near field can be done more efficiently.

The contributions of this paper are threefold. First, a far-field graphical model is built for adaptive far-field scene interpretation. Second, a dual-field sensing scheme is proposed to optimise the scene interpretation capability of the guidance systems. Third, a walking context estimation and message delivery strategy is designed to provide intelligent guidance service based on the dual-field sensing scheme.

## 2. Dual-Field Sensing

### 2.1. Dual-Field Sensing Scheme

**Figure 1:**
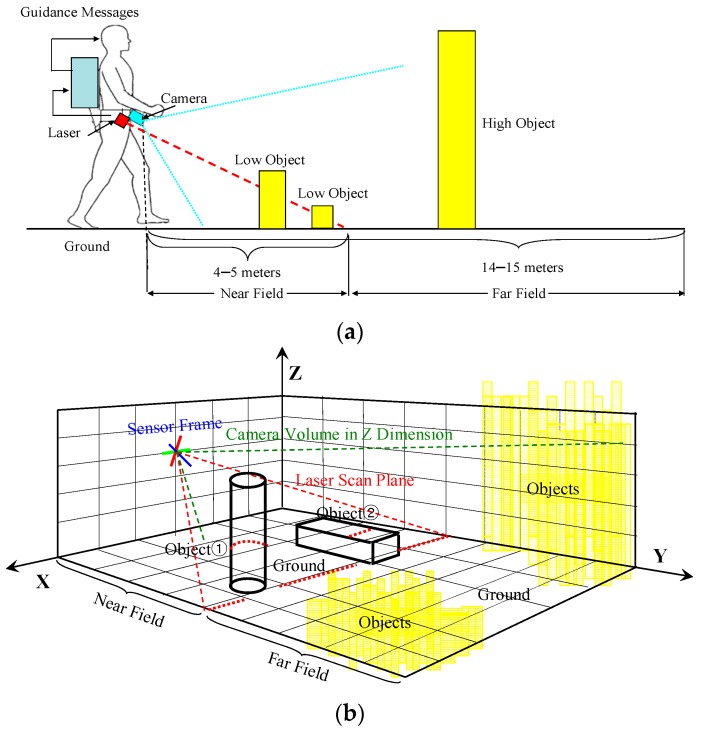
Dual-field sensing scheme. (**a**) Dual-field sensing scheme in 2D side view; (**b**) Dual-field sensing scheme in 3D frame.

**Figure 2:**
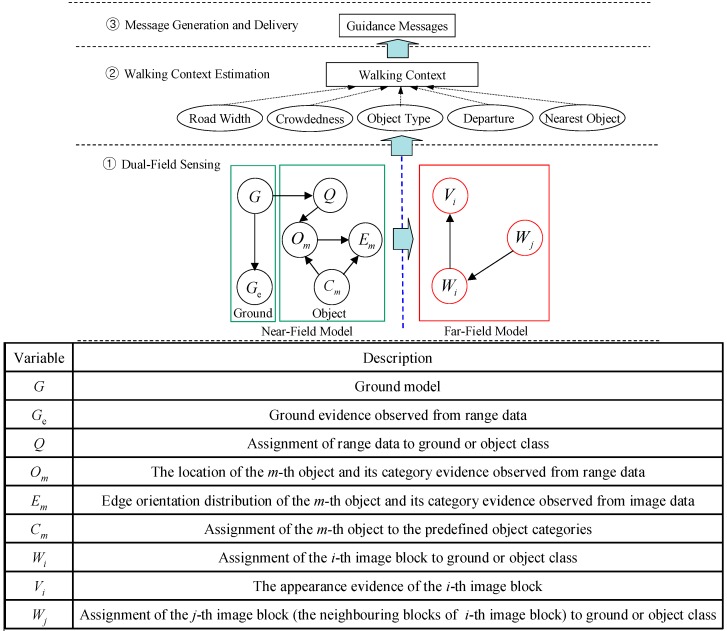
Guidance system model.

Figure 1a illustrates the dual-field sensing scheme in a 2D side view, and Figure 1b shows the dual-field sensing scheme in a 3D frame. In the proposed sensing scheme, a camera and a 2D laser scanner are fixed at a downward-pointing angle on the waist of a blind user. At this viewing angle, the combined sensors can cover a sufficient scale in the vertical dimension (Z axis), including the ground, low objects and high objects. The scale in the forward dimension (Y axis) is divided into near field and far field. The near-field range is covered by both the laser scanner and the camera, while the far-field range is covered only by the camera. In the horizontal dimension (X axis), both sensors can span a 120° view angle. The sensing data acquired from the scene are interpreted by a three-layer system model (Figure 2) on a portable computer carried by the blind user, and the interpretation results are delivered to the blind user in the form of guidance messages.

As shown in Figure 2, dual-field sensing is the most essential layer in the system model. It analyses raw sensor data and generates primitive knowledge about the scene for the upper layers. The primitive knowledge generally includes what objects are in the scene and where they are. As discussed in Section 1, there are several interpretation levels for this primitive knowledge, which we refer to as ‘scene interpretation resolution’. In our previous work, the interpretation resolution in the near field was upgraded to the high level, and blind users are happy with the increased knowledge about the status of each object in the near-field range. However, some misleading behaviours of the guidance system are encountered when the interpretation scale is confined to the near-field range. Therefore, it is necessary to include more knowledge by looking at the far-field area.

Unlike the high interpretation resolution in the near-field range, the interpretation resolution in the far-field area is set at the medium level. In the dual-field sensing scheme, the interpretation knowledge from the near field and the far field plays different roles in guidance tasks for blind people. The near field is a closer range on which more user attention is focused. Therefore, the near-field knowledge should be interpreted in high resolution and delivered to the user for advanced perception. In comparison, the far field is a more distant area that is not instantly related to the blind user. Therefore, far-field knowledge should contribute more to the overall context estimation compared to direct message delivery. In this sense, a medium interpretation level is sufficient for the far-field area.

As illustrated in Figure 1b, two different levels of interpretation resolution are set for the dual-field sensing scheme. In the near-field range that both the laser scanner and the camera can cover, a high resolution level is set to determine the status of each object and of the ground. In the far-field range that only the camera can cover, a medium resolution level is set to discriminate object groups from the ground.

### 2.2. Dual-Field Sensing Model

As shown in Figure 2, there are three layers in the proposed system model. The essential layer is the dual-field sensing layer, which is responsible for scene interpretation. The dual-field sensing layer is modelled using a probabilistic graphical model. In the near-field graphical model, the ground and each object’s state are jointly inferred by combining laser and image data. Random variables are defined to handle the uncertainties regarding ground location (*G*), each object’s location (*O_m_*), and each object’s type (*C_m_*). The probability distributions of these target random variables are estimated by observing a set of evidence distributions, such as ground fitting error (*G*_e_), range data assignment (*Q*) and edge orientation of an object (*E_m_*). Finally, an optimal inference about the ground and each object’s state is obtained by maximising the joint probability distribution of all random variables in the near-field graphical model.

**Figure 3:**
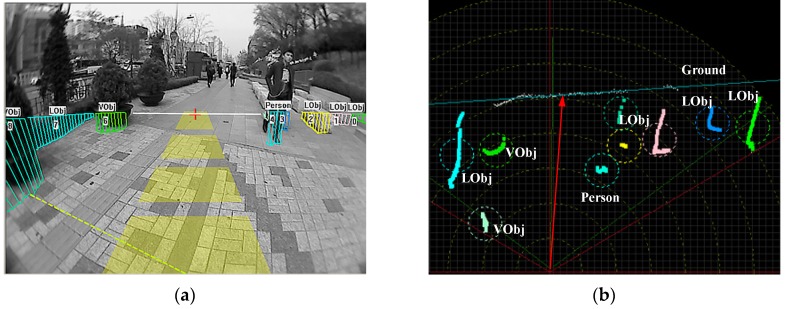
Near-field sensing scenario. (**a**) Near-field sensing result in the image domain; (**b**) Near-field sensing result in the range data domain.

Figure 3 shows a typical near-field sensing scenario. Basically, the range data from a 2D laser scanner are used as the main clue to infer the location of the ground and of each object. As shown in Figure 3b, a linear model (*G*) is adopted to approximate the ground in the scene. The proper ground line is found by maximising a fitting error distribution (*G*_e_). Based on the fitted ground line, an optimal assignment of range data to the ground or object is determined by maximising (*Q*). The object data points are further assigned to each individual object by searching for local maxima in a smooth likelihood function (*O_m_*).

To categorise generic objects on urban roads, a multimodal profile representation of an object is defined by integrating multiple cues from laser and edge profiles. Based on this multimodal profile model, generic objects on urban roads are categorised into seven prototypes. The most probable type of each object (*C_m_*) is determined by maximising the likelihood between the object’s multimodal evidence (*O_m_*, *E_m_*) and each category’s prototype $\left({\tilde{O}}_{k},{\tilde{E}}_{k}|k=1,2,\ldots 7\right)$. From the interpretation output of the near-field model, multiple cues are extracted to build an adaptive scene prototype that is cascaded into the far-field model using a cross-field interpretation strategy. This cascaded scene prototype is used as a basis to help infer the far-field image content in block units. In the far-field graphical model shown in Figure 2, the *i*-th image block is interpreted as the ground (*W_i_* = ground) or an object (*W_i_* = object) by observing its appearance evidence (*V_i_*) and its spatial evidence (*W_j_*) with respect to the cascaded scene prototype from the near-field. The optimal interpretation of the far field is decided by maximising the joint probability distribution of {*W_i_*, *V_i_*, *W_j_*} in the far-field graphical model. The far-field graphical model is discussed in detail in the following section.

## 3. Far-Field Scene Interpretation

### 3.1. Far-Field Graphical Model

To keep the sensing platform as simple as possible, no other sensors are added into our system apart from the downward-looking laser scanner and camera. Under this sensor configuration, only image data from a single camera are available for far-field scene interpretation.

There are two major challenges when using only image data in scene interpretation task. First, the road appearance encoded in image data tends to show large variability when blind people walk in different environments. Second, the lack of depth information in an image causes larger uncertainties when discriminating the ground from objects. To handle these two challenges, a far-field graphical model is proposed, as illustrated in Figure 2.

In the far-field interpretation, the image domain is divided into blocks as basic interpretation units, and the goal is to interpret each image block as either ground type (*W_i_* = Ground) or object type (*W_i_* = Object). Several clues can be exploited to infer (*W_i_*) in the far-field image domain. It turns out that the type of the *i*-th image block (*W_i_*) is related to its appearance property (*V_i_*) and to the types of its neighbouring blocks (*W_j_*). Therefore, the value of (*W_i_*) should be jointly inferred with (*V_i_*) and (*W_j_*). The joint probability distribution of {*W_i_*, *V_i_*, *W_j_*} can be derived as in Equation (1):

(1) $$\begin{array}{ll} P(W_{i},V_{i},W_{j}) & =P(V_{i}|W_{i},W_{j})P(W_{i},W_{j})\\ & =P(V_{i}|W_{i})P(W_{i}|W_{j})P(W_{j}) \end{array}$$

In Equation (1), P (*V_i_* | *W_i_*, *W_j_*) measures the probability that the appearance property (*V_i_*) occurs given its class type (*W_i_*) and its neighbouring block’s class type (*W_j_*). It is reasonable to assume that (*V_i_*) is conditional independent of (*W_j_*) given (*W_i_*). Therefore, P(*V_i_* | *W_i_*, *W_j_*) is equivalent to P (*V_i_* | *W_i_*). The probability term P (*W_j_*) P (*W_i_* | *W_j_*) captures how the confidence of the *j*-th block type P (*W_j_*) influences the confidence of the *i*-th block type P (*W_i_* | *W_j_*). Using the total probability theorem, a spatial constraint between P (*W_j_*) P (*W_i_* | *W_j_*) and P (*W_i_*) can be derived as in Equation (2), where (g) indicates the ground type label and (o) represents the object type label:

(2) $$P(W_{i}=g)=P(W_{i}=g|W_{j}=g)P(W_{j}=g)+P(W_{i}=g|W_{j}=o)P(W_{j}=o)$$

Substituting Equation (2) into Equation (1), P (*W_i_*|*W_j_*) P (*W_j_*) can be regarded as playing the role of a prior probability P(*W_i_*) that carries the spatial constraint, and P (*V_i_* | *W_i_*) serves as the likelihood that carries the appearance clue. The optimal interpretation of (*W_i_*) is finally determined by maximising the joint probability of {*W_i_*, *V_i_*, *W_j_*}, as in Equation (3):

(3) $$W_{i}=\underset{t,t^{\prime }\in \{\text{ground},\text{object}\}}{\text{arg}\;\text{max}}P(W_{i}=t,V_{i}=v,W_{j}=t^{\prime })$$

In the following sections, the probability distribution terms derived in Equation (1) will be defined, and the cross-field strategy used to calculate their values will be presented. In the near-field scene interpretation, the near-field graphical model provides reliable interpretation results by integrating range data with image data. In order to generalise the interpreted scene knowledge from the near field to assist the far-field scene interpretation, a cross-field interpretation strategy is designed to cascade an adaptive scene prototype from the near field to the far field. The cross-field strategy involves two aspects: cross-field appearance interpretation and spatial context propagation. The scene prototype that is cascaded by the cross-field strategy also includes appearance prototype and spatial context prototype. The basic assumption for the cross-field strategy is the consistency from the near field to the far field in terms of appearance and spatial properties.

### 3.2. Cross-Field Appearance Interpretation

#### 3.2.1. Appearance Prototype Building on Top-View Domain

**Figure 4:**
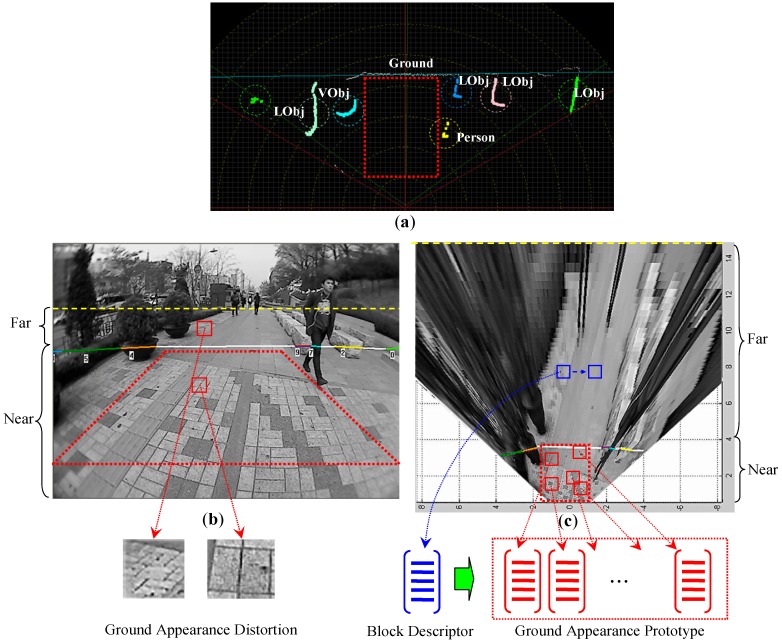
Cross-field appearance interpretation. (**a**) Block sampling ROI in the range data domain; (**b**) Block sampling ROI in the original image domain; (**c**) Appearance prototype building and matching on top-view image domain.

As shown in Figure 4, the appearance prototype of the ground is built by sampling image blocks from the near-field ground areas. Two aspects in this self-supervised sampling stage will influence the representation power of the appearance prototype. First, it is important that each sampled block contain correct information for the ground appearance. Therefore, the sampled image block must not be contaminated by the appearance content from objects. Second, the sampled image block should contain high descriptive content for the ground appearance. In practice, both of these two aspects can be achieved by choosing the correct sampling positions properly.

As shown in Figure 4a, a sampling region of interest (ROI) can be set based on the location of ground laser profiles in the range data domain. This sampling ROI is defined as the largest rectangular region where no object laser profiles appear. After mapping the ROI from the range data domain onto the image domain, the ROI corresponds to a consistent ground area in the image where no object contents are included. Figure 4b shows the mapped ROI in the original image domain. Therefore, by sampling image blocks inside the ROI on the image domain, the risk of including non-object appearance in the ground prototype can be avoided.

Inside the sampling ROI, feature points are detected using the Features from Accelerated Segment Test (FAST) [24] detector. To optimise the detection performance of the FAST detector, the decision tree of the detector is re-trained using ground images from our application environment. After the FAST detector is customised for the ground appearance in our application, it can detect corner-like feature points at very high speed. The FAST feature points generally indicate a highly descriptive neighbourhood with rich texture content. Therefore, the image blocks are sampled based on the FAST feature points to ensure inclusion of highly descriptive appearance content.

However, a problem exits in the original image domain that may cause the cross-field appearance interpretation to fail. In the original image, a distinct perspective effect can be observed in the ground area. As shown in Figure 4b, two red blocks with the same size are sampled from the ground area. The block from the near field contains two pavement bricks, while the block from the far field includes almost 10 bricks of similar size. This observation indicates that the pixel in the far-field image encodes a larger metric scale compared to the pixel in the near-field image. In addition, the shape of the pavement in the sampled block is also distorted from near field to far field. This shows that the perspective effect also alters the pixel distribution in a given block.

**Figure 5:**
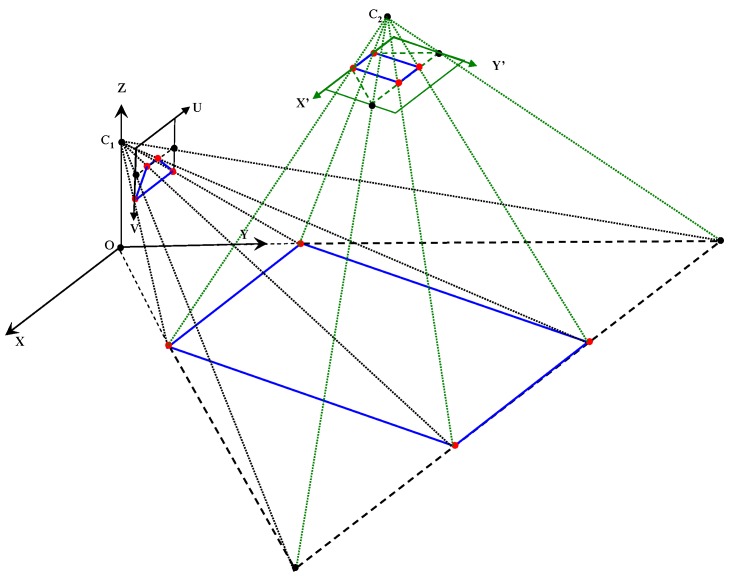
Perspective mapping and top-view mapping.

The perspective distortion observed in the ground area can be verified from the perspective mapping geometry, as illustrated in Figure 5. In Figure 5, **W** = {(*x*,*y*,*z*)} is the 3D world space, **I** = {(*u*,*v*)} is the 2D image plane. The perspective mapping from the ground plane **G** = {*x*,*y*,0} to the image plane **I** is defined by Equation (4), where (*h*) is the camera’s height above the ground, (*θ*) and (*γ*) are the camera’s viewing angles in the vertical and lateral directions, (2*α*) is the camera’s angular aperture and (*n*) is the camera’s resolution. Through this perspective mapping, the scales in X, Y and Z dimensions are not preserved when mapping onto the image plane **I**, which leads to perspective distortion.

(4) $$\left\{ \begin{array}{l} u(x,y,0)=\frac{\left(n-1\right)}{2\alpha }\cdot [\text{arctan}(\frac{h\text{sin}(\text{arctan}(y/x))}{y})-(\theta -\alpha )]\\ v(x,y,0)=\frac{\left(n-1\right)}{2\alpha }\cdot [\text{arctan}(y/x)-(r-a)] \end{array}\right.$$

The perspective distortion violates the consistency assumption of the cross-field strategy by making the far-field appearance significantly deviate from the near-field appearance. To solve this problem, inverse perspective mapping (IPM) [25] is used to remove the perspective distortion of the ground by remapping the original image to a top-view image plane. As illustrated in Figure 5, **T** = {(*x’*, *y’*)} is a top-view image plane that is parallel to the ground plane of the **W** space. The remapping from the original image plane **I** to the top-view image plane **T** is defined by Equation (5). Through this top-view mapping, the perspective distortions in ground appearance are compensated for, and the scales in X and Y dimensions of the ground plane are rectified as being uniform from near field to far field. Therefore, to better generalise ground appearance from near field to far field, the ground appearance prototype is built by sampling image blocks from the top-view image instead of the original image, as shown in Figure 4c.

(5) $$\left\{ \begin{array}{l} x\prime (u,v)=h\cdot \text{cot}(\theta -\alpha +u\frac{2\alpha }{n-1})\cdot \cos(\gamma -\alpha +v\frac{2\alpha }{n-1})\\ y\prime (u,v)=h\cdot \text{cot}(\theta -\alpha +u\frac{2\alpha }{n-1})\cdot \text{sin}(\gamma -\alpha +v\frac{2\alpha }{n-1}) \end{array}\right.$$

**Figure 6:**
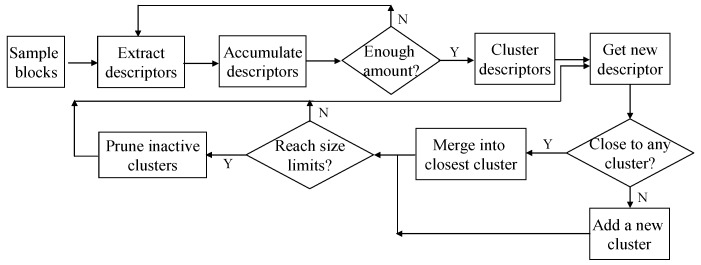
Adaptive appearance prototype building.

From the sampled image blocks, appearance descriptors are extracted for building the ground prototype on-line. As shown in Figure 6, the appearance descriptors extracted from the first few frames are accumulated in a feature pool. When the number of descriptors reaches a certain amount, the accumulated descriptors are clustered to determine a more general appearance pattern. The initial ground prototype is formed through this on-line clustering process. After that, the appearance prototype is incrementally updated by including new descriptors.

If the size of the appearance prototype reaches an upper limit, the less active clusters that have not been updated for a while will be pruned. This pruning scheme imitates short-term memory, which keeps the most recently observed appearance features in the prototype. Compared with near-field features that appeared a long time ago, the most recently observed near-field features are more likely to reappear in the coming far field.

To evaluate the activity of clusters in the appearance prototype, two indicators are associated with each cluster. One indicator is the latest time stamp when the cluster is updated with a new descriptor. The other is the number of new descriptors included in the cluster at the most recent unit time period. Based on these two active indicators, the activity of clusters can be evaluated. By using this on-line clustering and pruning scheme, the adaptive appearance prototype can be continuously updated to accommodate newly emerging information about the road appearance.

#### 3.2.2. Appearance Descriptor Extraction

**Figure 7:**
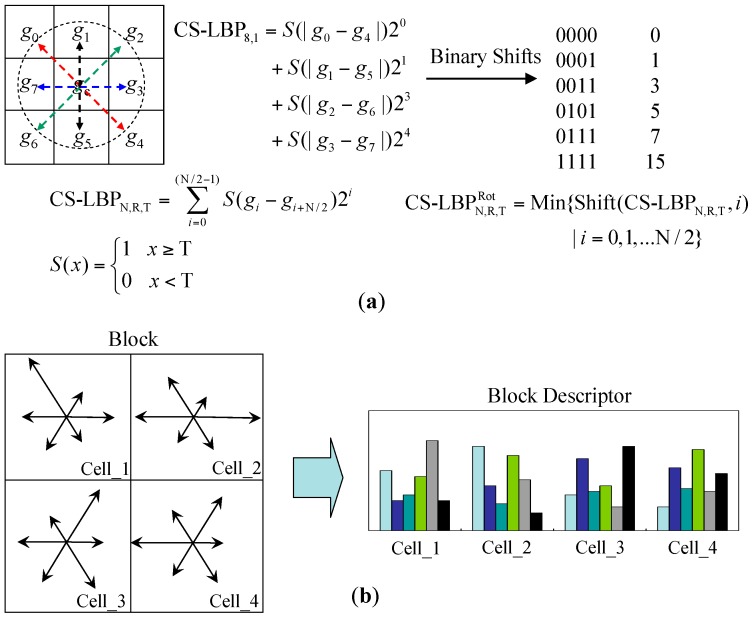
(**a**) Rotation-invariant CS-LBP descriptor; (**b**) Rotation-invariant CS-LBP block descriptor.

Appearance descriptors are extracted from the sampled image blocks in the top-view image to represent the block area with compact information. Two types of appearance descriptors are extracted, texture and colour descriptors. The texture descriptor is extracted using centre-symmetric local binary patterns (CS-LBP) [26]. As shown in Figure 7a, the CS-LBP operator compares the grey level of centre-symmetric pairs (*g_i_*) and (*g*_*i* + N/2_) that are located on a circle of radius (R). The grey level difference is encoded in four bits for an 8-neighbour region and then thresholded by a small value (T) to increase the robustness of the descriptor on flat image regions, where the grey value difference between pixels can be very small. This increased robustness on flat image regions makes CS-LBP very suitable for our interpretation task. In a local walking environment, it is often very likely that the ground appearance is dominated by flat and textureless areas.

In the ground appearance prototype, the CS-LBP is customised to serve the purpose of a texture template. The original CS-LBP descriptor is proposed for the purpose of matching exact key points between images with similar contents. The key-point detector will normalise the key-point supporting region to achieve invariance in scale and rotation for the CS-LBP descriptor. However, in our cross-field interpretation strategy, the goal is to get a dense labelling on every pixel in the far field rather than just a sparse set of key-point matches. Therefore, the key-point detector is dispensable in the cross-field strategy. Without the key-point detector, other solutions should be used to achieve scale and rotation invariance for the CS-LBP descriptor.

The IPM removes perspective distortion from the top-view image domain and normalises the scale of the ground plane along the X and Y dimensions. Therefore, by virtue of IPM, the scale invariance for the ground appearance can be achieved for the top-view image. To achieve rotation invariance, a simple solution based on binary shifts can be used. As shown in Figure 7a, the CS-LBP of an 8-neighbourhood region can be expressed using a 4-bit binary code in either clock-wise or counter clock-wise order. An interesting property of this binary code representation is that the rotation of the region can be mapped into binary shifts of the 4-bit code. Using this property, a neighbourhood region in an arbitrary orientation can be rotated to a canonical orientation by shifting the 4-bit code to its corresponding minimum or maximum value pattern. There are only six minimum-value patterns for a 4-bit binary code. The decimal values of these patterns can be used as an index for characterising rotation invariance of a particular neighbourhood region. The formula for calculating rotation-invariant CS-LBP is shown in Figure 7a, where Shift (*z*, *i*) indicates a binary shift of the 4-bit code (*z*) by *i* steps.

In the rotation-invariant CS-LBP descriptor, the texture statistic is captured by voting for a histogram with six bins based on each pixel’s CS-LBP value inside the region. Each bin corresponds to one of the six minimum value patterns that help achieve rotation invariance. To capture the spatial distribution of the texture statistics, a CS-LBP block descriptor is defined. As shown in Figure 7b, a CS-LBP block descriptor is composed of four cell descriptors that are spread in a Cartesian grid layout. Each cell descriptor is a histogram with six bins capturing the texture statistics inside the cell. Therefore, the block descriptor is a CS-LBP histogram with 24 bins. By capturing the spatial distribution of texture statistics, the CS-LBP block descriptor is more robust to partial illumination changes, such as shadows. To find the proper block size that achieves the best results, different combinations of block and cell sizes are tested on ground appearance data collected from the general urban environment. Experimental results show that the 16 × 16 block with an 8 × 8 cell size is best suited for our data.

In addition to the texture descriptor, a colour descriptor of the block region is extracted by concatenating an average hue, saturation and value (HSV) colour histogram with histograms of hue and saturation inside the sampling block. The colour histogram is obviously invariant to scale and rotation. Based on our experiment, the colour histogram is quantised to 16 bins for an acceptable balance between discrimination power and representation resolution. The combination of a rotation-invariant CS-LBP descriptor and an HSV colour descriptor constitutes the appearance descriptor that is used to build the appearance prototype.

#### 3.2.3. Cross-Field Appearance Prototype Matching

When the ground appearance prototype is built on-line, it grows into a compact representation of the ground appearance in the near field. The likelihood of having a ground image block in the far-field area can be calculated by matching the appearance evidence (*V_i_*) of the image block with the ground appearance prototype. A sliding window approach is used for this prototype matching process.

As shown in Figure 4c, on the top-view image, a 16 × 16 window slides across the far-field range with a stride of eight pixels. At each window position, the appearance descriptor (**H**) is extracted and matched with the ground prototype. The matching is performed by minimising the distance from (**H**) to each cluster centre ($\tilde{H}$) in the prototype. The distance is calculated using *X*^2^ distance, as in Equation (6), where (*H*_b_) and (*H*_c_) are the texture and colour descriptors extracted from the sliding window region, and (${\tilde{H}}_{b}$) and (${\tilde{H}}_{c}$) are the texture and colour descriptors of the cluster centre. The weight (*r*) assigns a bias towards the texture or colour descriptor.

(6) $$D(H,\tilde{H})=r\sum_{i=1}^{24}\frac{{\left(H_{b}^{i}-{\tilde{H}}_{b}^{i}\right)}^{2}}{2(H_{b}^{i}+{\tilde{H}}_{b}^{i})}+(1-r)\sum_{j=1}^{16}\frac{{\left(H_{c}^{j}-{\tilde{H}}_{c}^{j}\right)}^{2}}{2(H_{c}^{j}+{\tilde{H}}_{c}^{j})}$$

The scale and rotation are the major extrinsic changes that may obstruct the matching between (**H**) and ($\tilde{H}$) on the top-view image. As the invariance to scale and rotation has been achieved at the descriptor level, the distance $D(H,\tilde{H})$ can be used as a matching score that truly reflects the similarity between far-field appearance and the ground appearance prototype. Therefore, the ground likelihood P (*V_i_* | *W_i_* = ground) in the far-field graphical model is defined based on this distance, as in Equation (7):

(7) $$P(V_{i}|W_{i}=\text{ground})=\lambda e^{-\lambda \cdot D(H,\tilde{H})}$$

As in Equation (7), the ground likelihood is modelled using an exponential probability distribution function (PDF) with $D(H,\tilde{H})$ as the input random variable. In this exponential PDF, a larger distance value results in a smaller ground likelihood for the *i*-th image block in the sliding window. The parameter (*λ*) can be used to adjust the steepness of the PDF to map the distance values into probability values that are more properly scaled in the whole domain. The best match between the sliding window appearance and the ground prototype is found by minimising $D(H,\tilde{H})$. The minimisation process is performed using fast approximate nearest neighbour searches [27] in the ground prototype space.

### 3.3. Cross-Field Spatial Context Propagation

In the cross-field appearance interpretation process, the type of each image block is inferred independently using its appearance property alone, without considering the interpretation consistency with its neighbouring blocks. However, it can be observed that a particular type of region usually covers many adjacent image blocks which should be interpreted as the same type. The strong type consistency among neighbouring blocks in every direction constitutes a distinct spatial context inside a particular type of region. On the other hand, the type consistency will be attenuated in a particular spatial pattern at the boundary between two different types of regions. Therefore, the type consistency pattern among neighbouring blocks can be exploited as a complement to the block-specific appearance interpretation.

#### 3.3.1. Spatial Distribution Properties in the Top-View Domain

**Figure 8:**
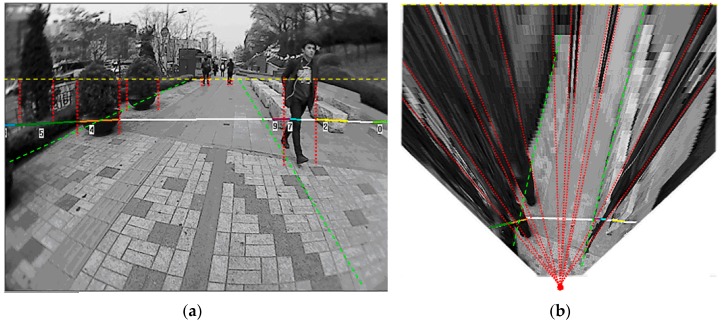
(**a**) Spatial distribution of the ground and object region in the original image domain; (**b**) Spatial distribution of the ground and object region in the top-view domain.

As shown in Figure 8b, some distinct spatial properties can be identified from the distribution of ground and object regions in the top-view domain. First, the ground usually corresponds to a large consistent area that spans the middle of the image domain. Second, the objects that have vertical boundaries in the original image are mapped to slanted regions that are stretched along the radial directions in the top-view image. The radial orientations of the object regions are greater than 90° in the left half of the image and are smaller than 90° in the right half of the image.

The radial distribution of the object regions can be verified using the IPM rules in Equation (5). The vertical object boundary in the **I** space can be represented by (*v* = *k*), where (*k*) is a constant value. This can be substituted into Equation (5) and obtain Equation (8), where the tangent term is a constant. This derivation implies that IPM maps vertical object boundaries into lines that pass through the origin point O, which corresponds to the camera centre’s perpendicular projection on the ground plane. Therefore, in the top-view image, the vertical surfaces of the object appear as slanted regions that are stretched along the radial direction with respect to the origin point O’s projection in the top-view plane.

(8) $$y=\text{tan}(r-\alpha +\frac{2\alpha k}{n-1})\cdot x$$

**Figure 9:**
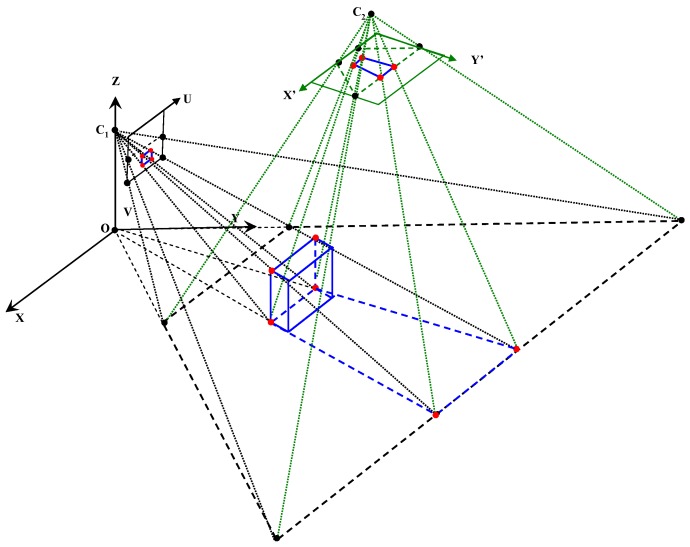
Spatial distribution properties of ground and object regions in perspective mapping and inverse perspective mapping.

The spatial interactions between the ground and the object can also be observed in Figure 8b. For objects that are located on the side of the road, their radially stretched regions will appear to the left or right side of the ground region. For an object that appears in the middle of the road, the object region will occlude parts of the ground region covering from the top of the image domain down to the object’s feet position. Therefore, it is less likely for an object region to appear in a six o’clock direction from a ground region in the top-view domain. These spatial distribution properties can be verified from the perspective mapping and IPM mapping models that are illustrated in Figure 9.

The above spatial distribution properties will generate specific patterns of type consistency among neighbouring image blocks in the top-view domain. The type consistency pattern in the neighbourhood of an image block is defined as the spatial context. In the following sections, a method using a neighbourhood histogram is proposed to build the spatial context prototype from the near-field interpreted data, and then the spatial context prototype will be propagated in the far-field image domain to refine the interpretation.

#### 3.3.2. Spatial Context Prototype Building

**Figure 10:**
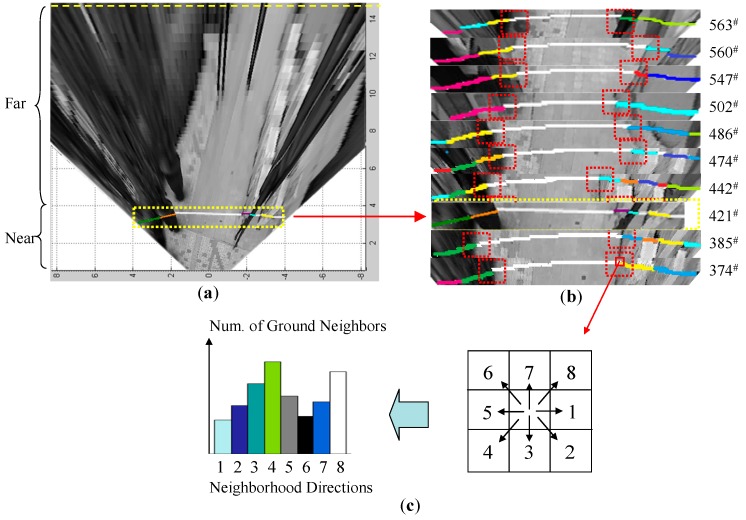
Learning spatial distribution properties from near-field labelled data. (**a**) Near-field ROI snapshot containing interpreted data; (**b**) ROI snapshot buffer; (**c**) Neighbourhood histogram voting.

The spatial context prototype among neighbouring image blocks is built on line from the near-field interpreted data. As shown in Figure 10a, a near-field ROI snapshot containing the interpreted image data is stored in a snapshot buffer. Each ROI snapshot records the image data that has been interpreted by the near-field graphical model.

As shown in Figure 10b, a set of buffered ROI snapshots is listed with their corresponding frame numbers. The ground pixels are labelled in white, and the object pixels are labelled in all other colours. At each ground (or object) pixel location, the pixel labels on its 8-neighbourhood directions are scanned, and the number of ground pixels in each direction is recorded in a neighbourhood histogram. After scanning all the labelled ROI images in the buffer, the neighbourhood histogram is able to capture the general spatial context in the current local scene. When a new ROI snapshot arrives, the neighbourhood histogram can be updated to reflect the new distribution pattern. To boost this on-line scanning process, the near-field pixels are grouped into a 5 × 5 block as a scanning unit. This scanning strategy helps to achieve a balance between scanning speed and spatial resolution.

**Figure 11:**
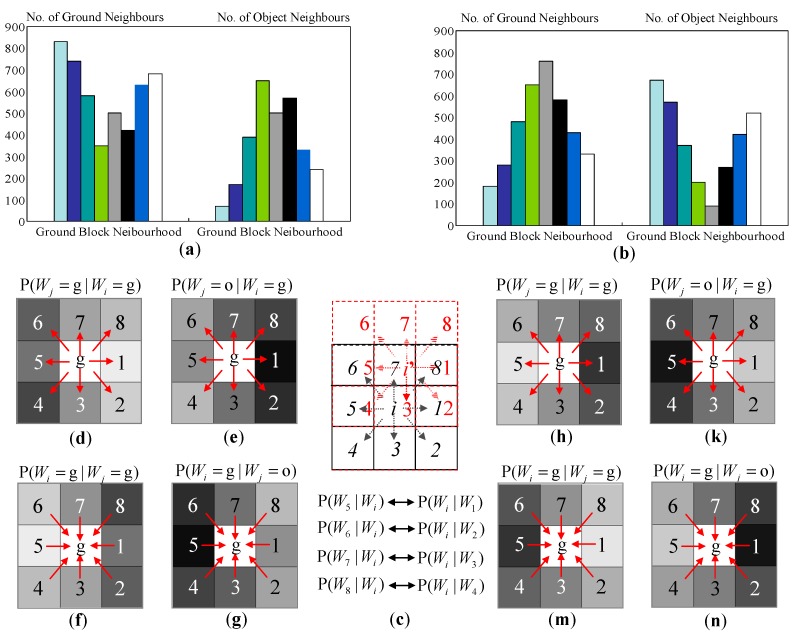
Spatial context prototype built from a neighbourhood histogram. (**a**) Neighbourhood histogram for the left half of the image; (**b**) Neighbourhood histogram for the right half of the image; (**c**) Transfer of neighbourhood dependency relations; (**d**–**g**) Spatial context prototypes for the left half of the image; (**h**–**n**) Spatial context prototypes for the right half of the image.

The neighbourhood histograms that are built from the above scanning process are shown in Figure 11a,b. Because the radial orientations of the object regions show a distinct divergence from the left half of the image to the right half of the image, this property will generate spatial contexts that are significantly different in the left half and the right half image. Therefore, two sets of neighbourhood histograms are built for the left half of the image and the right half of the image. The neighbourhood histogram captures the distribution of ground blocks and object blocks around a ground block neighbourhood.

Using the neighbourhood histogram of ground block distribution, the conditional probability P (*W_j_* = g | *W_i_* = g) can be calculated as in Equation (9), where *n_j_* is the *j*-th bin value of the neighbourhood histogram, and *N*_g_ is the total number of ground blocks that are scanned.

(9) $$P(W_{j}=g|W_{i}=g)=\frac{n_{j}}{N_{g}}$$

The conditional probability P (*W_j_* = o | *W_i_* = g) can be calculated in a similar way using the neighbourhood histogram of the object block distribution. The values of these conditional probabilities are shown in Figure 11d,e for the left half of the image and in Figure 11h,k for the right half of the image. The conditional probability P(*W_j_* = g | *W_i_* = g) reflects the probability of having a ground block at the *j*-th neighbouring position of a ground centre block. However, this is not the conditional probability that captures the type consistency influence from the neighbouring blocks to the centre block. The conditional probability that reflects the constraint from the neighbouring blocks (*W_j_*) to the centre block (*W_i_*) should be P (*W_i_* = g | *W_j_* = g), which is the inverse dependency version of the obtained P (*W_j_* = g | *W_i_* = g). By using the interrelation structure of the centre block and the neighbouring block, P (*W_i_* = g | *W_j_* = g) can be obtained based on P (*W_j_* = g | *W_i_* = g) by transferring the dependency order between *W_j_* and *W_i_*.

As shown in Figure 11c, supposing P (*W_i_* = g | *W*_7_ = g) is the probability to be computed, it describes the influence from the 7-th neighbouring block being interpreted as ground to the *i*-th centre block being interpreted as ground. If the centre block (*i*) of P (*W_j_* = g | *W_i_* = g) in Figure 11d is shifted to the 7-th block’s position (*i*’) in Figure 11c, then in the shifted neighbourhood, P (*W*_3_ = g | *W_i_*_’_ = g) actually describes the probability that P (*W_i_* = g | *W*_7_ = g) indicates. Therefore, by shifting the centre block of the P (*W_j_* = g | *W_i_* = g) to all the neighbouring block positions, a direct mapping relationship between P (*W_j_* = g | *W_i_* = g) and P (*W_i_* = g | *W_j_* = g) can be formed, as shown in Figure 11c. Using this mapping relationship, the value of P (*W_i_* = g | *W_j_* = g) at each neighbourhood position is obtained. In a similar way, P (*W_i_* = g | *W_j_* = o) can be obtained from P (*W_j_* = o | *W_i_* = g).

**Figure 12:**
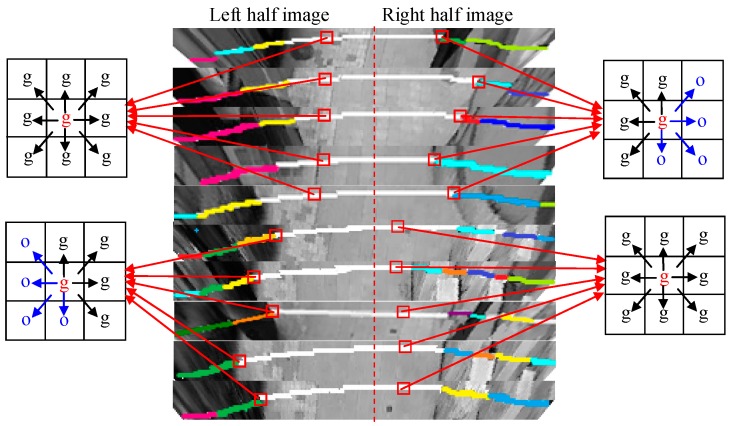
Spatial context comparison for the left half and the right half of the image.

The spatial context prototypes shown in Figure 11 are built from the near-field snapshot data shown in Figure 10b. It can be observed that the spatial context prototypes are able to capture the spatial distribution properties discussed in Section 3.3.1. As shown in Figure 12, when the ground block neighbourhoods of the consistent ground area are scanned, the spatial confidence in observing a ground block in each of the eight directions is high. However, this confidence will be attenuated when the intersection areas of the ground and the object are scanned. Due to the radial distribution of object regions in the top-view domain, the object regions are dominated by left diagonal structures in the left half of the image and by the right diagonal structures in the right half of the image. Therefore, in the left half, the spatial confidence in observing a ground block in the lower left diagonal directions will be attenuated, while in the right half image, the spatial confidence in observing a ground block in the lower right diagonal directions will be attenuated. In addition, if there are many object feet appearing in the near-field scene, then the spatial confidence in observing a ground block in the upper directions will be even more attenuated compared to the spatial confidence in observing a ground block in the lower directions.

The spatial context prototypes can be updated on-line using the latest near-field snapshots. This makes it possible to track the spatial changes in the local scene. The changes in spatial distribution of ground and objects will result in corresponding changes in the spatial context pattern. For example, if the blind user enters an open space from a very narrow and crowded scene, then the ground confidence in every neighbourhood direction will be increased compared to the old spatial prototype.

#### 3.3.3. Spatial Context Propagation

Based on the cross-field consistent assumption, the spatial distribution of the ground and objects in the far field may share some consistent spatial contexts with the near field. Therefore, the spatial context prototypes built from the near field are propagated to the far field to reduce interpretation ambiguities. During cross-field spatial context propagation, the top-view image is divided into the left domain and the right domain, each of which is divided into 5 × 5 block units. The spatial context prototype built from the left images is propagated in the left domain, and the prototype built from the right images is propagated in the right domain.

At the *i*-th image block, the spatial confidence in interpreting it as ground by observing the *j*-th neighbouring block can be expressed as in Equation (2), where the conditional probabilities P (*W_i_* = g | *W_j_* = g) and P (*W_i_* = g | *W_j_* = o) are applied from the spatial context prototype. By considering the spatial confidences received from all eight neighbouring blocks, Equation (2) can be expanded into Equation (8), where *r_j_* is a normalised weight factor representing the relative importance of each neighbourhood direction. In a general case, *r_j_* is simply assigned to 1/8 for each *j*-th direction.

The probability P (*W_j_*) in Equation (10) indicates the confidence in interpreting the *j*-th image block as ground or object. To give an initial estimate for this confidence, the ground appearance likelihood of the *j*-th image block is used for measuring P (*W_j_*). Finally, the spatial confidences from all the neighbouring blocks are applied to the ground appearance likelihood of the *i*-th image block, which constitutes the whole joint probability distribution of {*W_i_*, *V_i_*, *W_j_*} in the far-field graphical model. The final integration of cross-field likelihood and spatial context is expressed as in Equation (11), where P(*V_i_*|*W_i_*=g) defines the cross-field appearance likelihood and the reaming part defines the cross-field spatial context.

(10) $$P(W_{i}=g)=\sum_{j=1}^{8}r_{j}\cdot \sum_{W_{j}}P(W_{i}=g|W_{j})P(W_{j})$$

(11) $$P(W_{i}=g,V_{i},W_{j})\propto P(V_{i}|W_{i}=g)\cdot [\sum_{j=1}^{8}r_{j}\sum_{W_{j}=\{g,o\}}P(W_{i}=g|W_{j})P(W_{j})]$$

**Figure 13:**
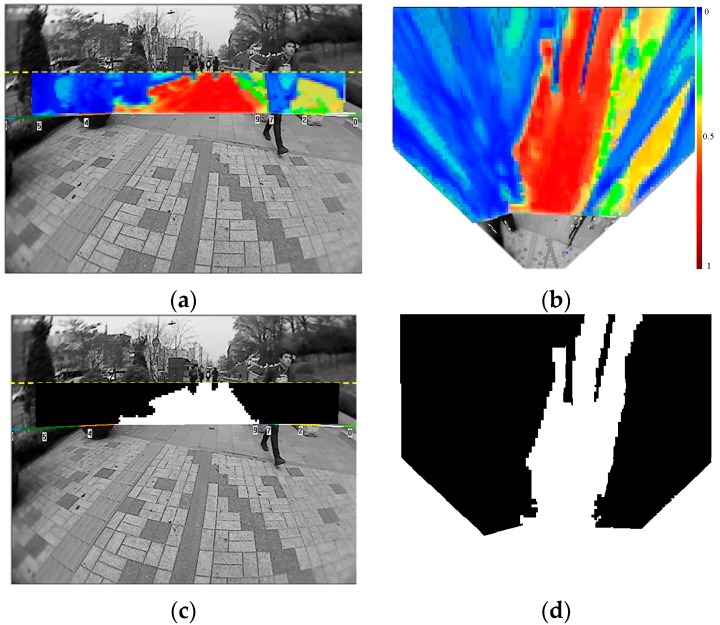
(**a**) Far-field ground probability map on the original image; (**b**) Far-field ground probability map on the top-view image; (**c**) Far-field scene interpretation on the original image; (**d**) Far-field scene interpretation on the top-view image.

An example of the far-field scene interpretation is shown in Figure 13. In Figure 13a,b, the probability value of P (*W_i_* = g, *V_i_*, *W_j_*) is encoded in heat colours. Warm colours indicate the high probability of being ground and the cold colours represent the low probability of being ground. The far-field scene interpretation is done on the top-view image domain using the cross-field scheme, and Figure 13) shows the mapping of P (*W_i_* = g, *V_i_*, *W_j_*) to the original image domain. Due to the radial distortion of the wide-angle lens in our on-board camera, the regions close to the borders of the original image cannot be mapped onto the top-view plane. Therefore, there are no corresponding probability values for the left and right border regions of the original image.

The final interpretation of the *i*-th image block is made by maximising the joint probability distribution P (*W_i_*, *V_i_*, *W_j_*), as in Equation (3). If P (*W_i_* = g, *V_i_*, *W_j_*) > P (*W_i_* = o, *V_i_*, *W_j_*), then the image block is interpreted as a ground type. Otherwise, it is interpreted as an object type. The final interpretation result is shown in Figure 13d, where ground blocks are labelled in white and object blocks are labelled in black. Figure 13c shows the mapping of the interpretation result from the top-view domain back to the original image domain.

## 4. Dual-Field Sensing Fusion

### 4.1. Dual-Field Sensing Map

In the dual-field sensing framework, the near-field model provides essential interpretation in high resolution, and the far-field model extends this interpretation scale based on a cross-field interpretation scheme. By integrating the essential interpretation in the near field with the extended interpretation in the far field, a dual-field sensing map can be generated. In the guidance system model shown in Figure 2, the dual-field sensing map plays the role of an interface between the dual-field sensing layer and the upper layers.

**Figure 14:**
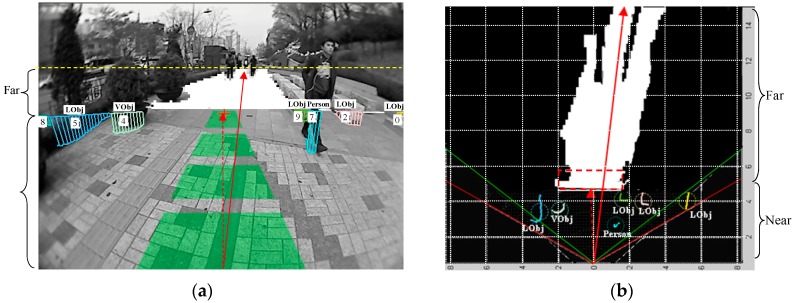
(**a**) Dual-field sensing map on the original image; (**b**) Dual-field sensing map from the top-view domain.

The dual-field sensing map is built as a 2D grid map, as shown in Figure 14b. The sensors are located at the origin point (0, 0), the laser’s scanning range spans the pair of red line borders and the camera’s viewing range is marked out by the green line borders. The horizontal and vertical axes span an observer coordinate frame in parallel to the ground. In the near-field range, the ground and object profiles in the laser’s scanning frame are mapped onto the grid map by multiplying the cosine of the angle between two frame planes. In the far-field range, the interpreted top-view image is mapped onto the grid map using the camera’s intrinsic and extrinsic parameters. Figure 14a shows the dual-field sensing map in the original image view.

The dual-field sensing map distinctly improves the guidance system performance compared with that using only the near-field sensing map. Specifically, the efficiency of the safe direction guidance is improved a lot. In Figure 14b, the dashed arrow shows a safe direction that is found by following the centre of the ground laser profile on the near-field sensing map. In comparison, the solid arrow shows a safe direction that is found by casting polar rays on the dual-field sensing map. It is obvious that the solid arrow gives more efficient guidance by virtue of the extended interpretation scale of the far-field range. In the following section, the advantages of using dual-field sensing for walking context estimation and guidance message delivery will be discussed.

In real applications, the dual-field sensing scheme provides adaptable performance based on different local conditions. In local areas where there is good consistency from near field to far field, the dual-field sensing is fully operational to maximise guidance performance and user experience. However, a large inconsistency between near-field and far-field appearances may cause temporary failure in far-field interpretation. To identify such failures, the interpretation result inside a rectangular region just ahead of the ground laser segment is monitored, as illustrated by the blue rectangle in Figure 14b. If over 80% of the area inside this region is interpreted as object type, then either there is a large object right ahead, or there is a large change in road appearance. In this case, the far-field model is temporarily disabled to wait for the cross-field prototype to be updated, and the system relies entirely on the near-field model to provide essential interpretation for the guidance task.

### 4.2. Walking Context Estimation Based on Dual-Field Sensing

**Figure 15:**
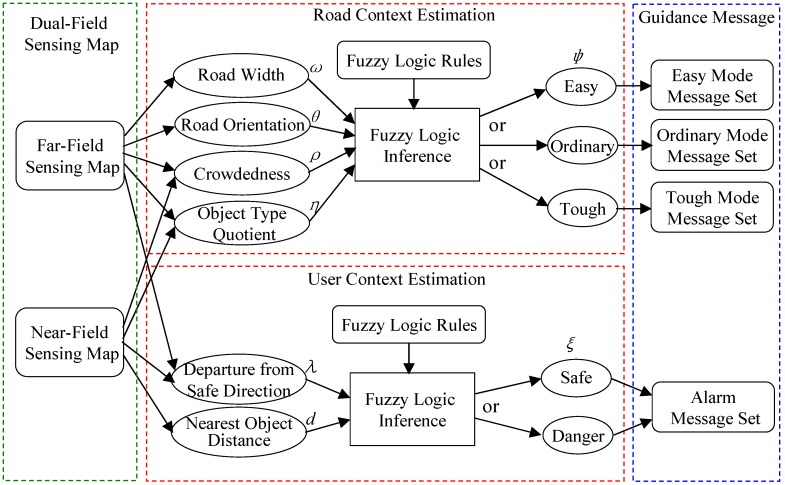
Walking context estimation based on dual-field sensing.

The dual-field sensing scheme also brings benefits to its upper layers in the guidance system model, as shown in Figure 15. The walking context layer is introduced into the guidance system as a middle layer between the dual-field sensing layer and the guidance message layer. The purpose of having the walking context layer is to achieve a more efficient message delivery mechanism.

The dual-field sensing layer offers large volumes of interpreted scene information to be delivered to the blind user in the form of audio or tactile modalities. However, if the raw interpretation from the sensing layer is forwarded to the blind user all at once, it may easily cause message overload for the user’s perception. To solve this problem, the walking context layer is proposed to evaluate the overall road condition (road context) and the interaction between the blind user and the road environment (user context). Based on the estimated walking context, the importance of messages to the blind user in a certain walking context can also be evaluated. Finally, the messages are delivered to the blind user according to their importance in the current walking context.

As shown in Figure 15, the walking context includes the road context and the user context. The road context is defined as a variable (*ψ*) that evaluates the degree of difficulty of walking along the road. The value of (*ψ*) ranges from 0% to 100%, and three fuzzy sets are defined to describe this difficulty level in terms of ‘easy’, ‘ordinary’ and ‘tough’. To infer the value of (*ψ*), several evidence measurements are collected from the dual-field sensing map, and a set of fuzzy logic rules are defined to relate these evidence measurements to the road context status.

**Figure 16:**
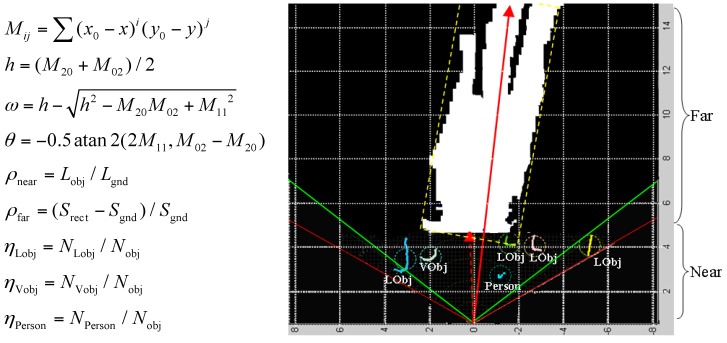
Definition of walking context measurements.

The measurements used to infer the road context are defined in Figure 16. The road width (*ω*) and orientation (*θ*) are estimated using the geometric moments (*M_ij_*) of the ground region, which can be calculated using the region’s boundary points (*x*,*y*) and the region’s centre of mass (*x*_0_, *y*_0_). By using the geometric moments, the smallest bounding rectangle can be fitted to the ground region boundary. The far-field crowdedness (*ρ*_far_) is thus defined based on the area difference between the bounding rectangle (*S*_rect_) and the inner ground region (*S*_gnd_). Meanwhile, the near-field crowdedness (*ρ*_near_) is calculated using the ratio between the total length of the object laser profile (*L*_obj_) and the length of the ground laser profile (*L*_gnd_). The object type quotient (*η*) measures the ratio of the number of each object type (*N*_Lobj_, *N*_Vobj_, *N*_person_) within the total number of objects *N*_obj_.

**Figure 17:**
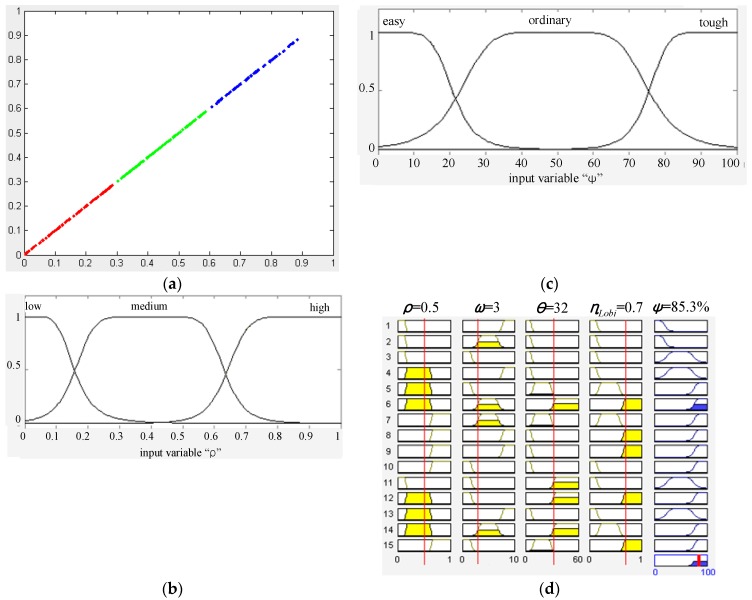
(**a**) Clustering of the crowdedness measurements; (**b**) Fuzzification of the crowdedness measurement (ρ); (**c**) Fuzzification of the road context (ψ); (**d**) Fuzzy logic inference of road context using evidence measurements and fuzzy rules.

In order to use fuzzy logic rules to infer the road context, the evidence measurements {*ω*, *θ*, *ρ*, *η*} and road context (*ψ*) need to be mapped to fuzzy sets from their real-valued domains using membership functions. The fuzzification of the road context (*ψ*) is shown in Figure 17c, where a bell-shape membership function is used to map the value of (*ψ*) to the three fuzzy sets. The parameters of the bell-shape function are specified according to empirical data and user experience. The fuzzification of the evidence measurements is performed by observing the data distribution in their original real-valued domains. As shown in Figure 17a, the crowdedness measurements (*ρ*) from various scenarios are collected and gathered into three clusters. Based on the three data clusters, three fuzzy sets (low, medium, high) and corresponding membership functions are defined, as shown in Figure 17b. After fuzzification of all the measurement variables, a set of fuzzy logic rules can be defined in the form of ‘If (*ρ* is low) and (*ω* is wide) and (*θ* is small) and (*η_Lobj_* is low), then (*ψ* is easy)’. Using the fuzzy logic rules, fuzzy logic inferences based on min and max operations can be performed to infer the value of (*ψ*) from a specific set of evidence measurements, as shown in Figure 17d.

The user context (*ξ*) is defined as the degree of safety in regard to the user’s interaction status with the road environment. The value of (*ξ*) can also be inferred from the two evidence measurements (*λ*) and (*d*) by using fuzzy logic inference. The measurement of departure (*λ*) includes two components: departure from the dual-field safe direction (*λ*_dual_) and departure from the near-field safe direction (*λ*_near_). The measurement (*λ*_near_) is used instead of (*λ*_dual_) in case the far-field interpretation fails. The departure (*λ*) is measured based on the angle that is formed between the forward axis of the sensor frame and the estimated safe directions. The measurement of the nearest object distance (*d*) is collected directly from the range data profile of the nearest object.

### 4.3. Context-Aware Guidance Based on Dual-Field Sensing

The dual-field sensing layer and the walking context layer provide a solid basis for the guidance message layer. Using the information obtained from the lower layers, a message delivery scheme is designed, as shown in Table 1. In order to optimise the delivery throughput, the messages are delivered in multiple modalities. Essential messages are delivered using an acoustic beeper or tactile arrays, and the context messages are delivered in the form of verbal instructions. In this way, the essential messages and context messages can be delivered in parallel without interfering with each other.

The delivery timing is different for different types of messages to optimise the user’s perception experience. As shown in Table 1, the essential messages are delivered continuously to track the status of nearby objects in real time. The safe direction and alert messages are triggered by specific events. The safe direction message is triggered when there is sufficient change in the safe direction. The alert messages are triggered when the user context (*ξ*) reaches a predefined threshold in the ‘danger’ status.

**Figure 18:**
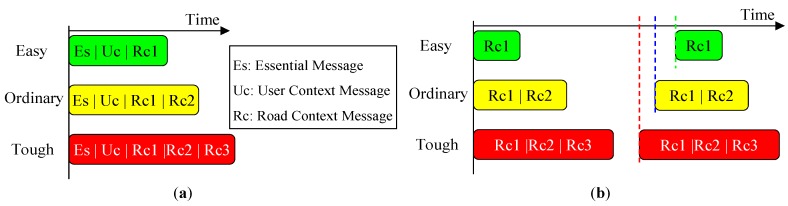
(**a**) Message set definition in different road contexts; (**b**) Delivery timing of road context message set in different road contexts.

The road context messages are delivered periodically in a frequency that is controlled by the road context status (*ψ*). As shown in Figure 18b, the delivery interval is inversely proportional to the value of (*ψ*). Meanwhile, a message set is defined as a delivery unit for each period. In Figure 18b, ‘Rc1’ corresponds to the message (*ψ*) in Table 1, and ‘Rc2’ and ‘Rc3’ indicate the two messages that are selected from the messages {*ω,θ,ω,η*}. The selection criterion is based on their saliency in the current road context.

The saliency of context messages in different road contexts is defined in Table 2 and is summarised from the fuzzy logic rules in the road context inference. The message saliency is divided into three levels in each context mode, and the saliency order is: S1 > S2 > S3. For messages within the same level, their saliency is evaluated by the fuzzified values of their corresponding measurement. The message that has a higher fuzzified value is more salient than the others. The messages that have not been selected in the initial period have higher priority to be delivered in the next period.

The above saliency-based delivery scheme can be illustrated using the case in Figure 17d. The fuzzy inference result of the case in Figure 17d shows that the road context (*ψ*) is in ‘tough’ status. The fuzzy logic rule that contributes the most to this inference result is rule No. 6. According to rule No. 6, the context messages to be delivered can be identified as (*ρ.*medium, *ω*.normal, *θ.*high, *η*.high). According to Table 2, the messages (*θ.*high) and (*η*.high) are more salient than (*ρ.*medium) and (*ω*.normal). Furthermore, it can be noticed that (*η*.high) has larger membership values than (*θ.*high) and (*ρ.*medium) has larger membership values than (*ω*.normal). Therefore, the final saliency order in this case is (*η*.high > *θ.*high > *ρ.*medium > *ω*.normal), and (*η*.high) should be selected as R2 and (*θ.*high) should be selected as R3. In the next delivery period, the messages concerning (*ρ*) *and* (*ω*) that have not been delivered in the previous period will gain extra weight in terms of saliency.

When messages of different types are prompted in the same moment, a proper priority scheme needs to be defined to resolve the conflict. As shown in Figure 18a, the event-triggered message types have higher priority than the periodically delivered messages. Therefore, the event-triggered messages can interrupt the periodic message set when conflict occurs. During message delivery, the timeslots will be allocated to the event-triggered messages in priority of order when they are triggered, and the periodic messages concerning road context are sent out using the remaining timeslots.

## 5. Experimental Results

### 5.1. Interpretation Performance of the Far-Field Model

To test the performance of far-field scene interpretation, the test data are collected using the same sensor platform as in [23]. The sensor platform is mounted on a human body, and the image frames are recorded while walking along various pedestrian paths.

**Figure 19:**
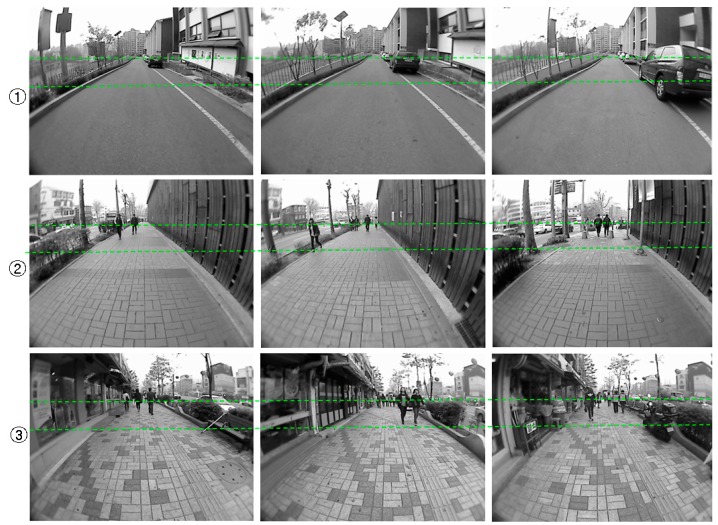
Sample images from the test scene.

The example images collected from the three test scenes are shown in Figure 19. The complexity of the road condition increases from scene ① to scene ③. The image areas between the two dashed lines correspond to the far-field area that needs to be interpreted. In the experiment, the image frames are sampled every three seconds to allow enough variance in the evaluation set. Finally, one hundred image frames from each test scene are selected for the experiment. The ground and object areas in these evaluation image frames are manually labelled as ground truth data.

**Figure 20:**
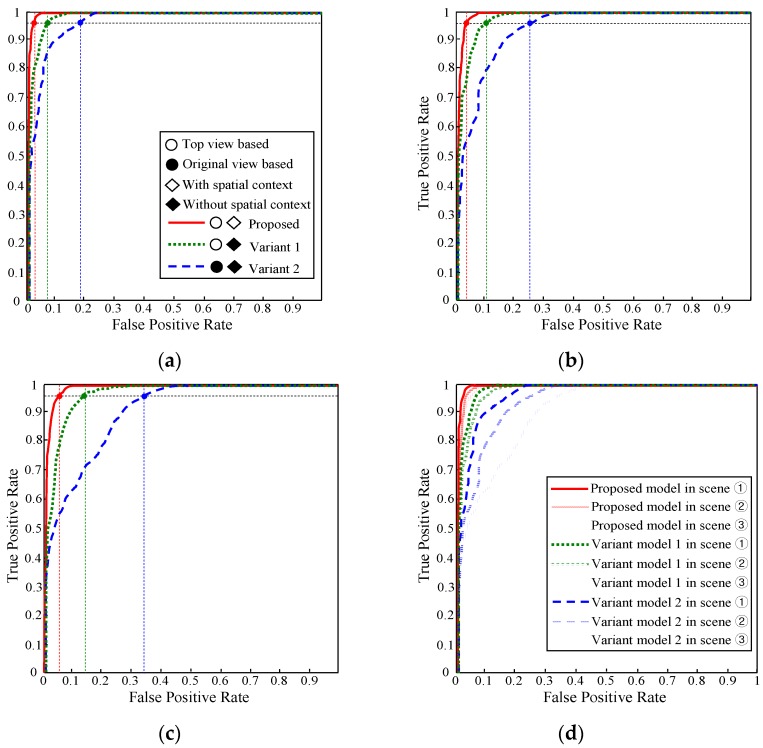
(**a**) Far-field model performances in test scene ①; (**b**) Far-field model performances in test scene ②; (**c**) Far-field model performances in test scene ③; (**d**) Far-field model performances in all three test scenes.

During the experiment, the appearance and spatial prototypes are built on-line using the near-field interpreted data in the preceding frames of each evaluation image frame. Next, the far-field image data in each evaluation image frame are interpreted using the built prototypes based on the far-field graphical model. As a result, a far-field ground probability map P (*W_i_* = g, *V_i_*, *W_j_*) is obtained for each evaluation image frame. The decision rule ‘P (*W_i_* = g, *V_i_*, *W_j_*) > U’ is used to interpret an image block as ground. For each specific value of (U), the interpreted ground areas in the evaluation image frames are compared with the ground truth data to calculate the average true positive (TP) rate and false positive (FP) rate. After the average TP and FP rates are calculated at a specific decision threshold (U), the decision threshold (U) is varied to get other combinations of TP and FP rates. Finally, the receiver operating characteristic (ROC) curves of each test scene are obtained, as shown in Figure 20.

The ROC curves in Figure 20 show the interpretation performance of three far-field models. The red solid curve represents the proposed far-field model that uses a top view-based appearance interpretation and spatial context propagation. The green dotted curve shows a variant model 1 that uses a top view-based appearance interpretation without spatial context propagation. The blue dashed curve corresponds to a variant model 2 that uses a perspective view-based appearance interpretation without spatial context propagation. Based on the ROC curves, the variant model 2 performs the worst. This is mainly due to the trouble caused by perspective distortion of the prototype building and matching of local texture descriptors. The variant model 1 that uses top view-based prototype building and matching has a remarkable performance gain over the variant model 2. The effect of the spatial context propagation is verified by comparing the red solid curve to the green dotted curve. The extra performance gain of the proposed model shows that the special context helps to remove many spatially inconsistent errors caused by local appearance descriptor matching.

The proposed far-field model also shows stable performance under different road conditions. As shown in Figure 20a–c, the performance can be evaluated using the variation of the FP rate of the three models at a fixed TP rate of 96% across the three test scenes. The FP rate of the proposed model only degrades from 3.1% in test scene ① to 5.6% in test scene ③. However, the FP rate of the variant model 1 increases from 8.2% to 14.7%, and the FP rate of the variant model 2 increases from 18.8% to 34.5%. The performance variation across the three test scenes can be more easily observed in Figure 20d, where the ROC curves of the three test scenes are drawn onto the same graph. It shows that the proposed model maintains a low FP rate consistently at a high TP rate under different road conditions.

**Figure 21:**
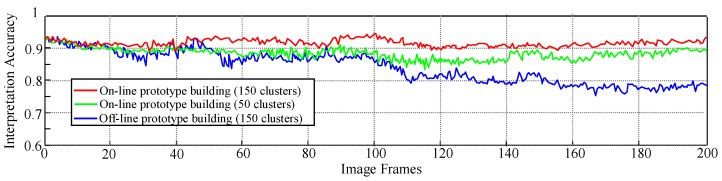
Far-field interpretation accuracy for different prototype building mechanisms.

Figure 21 shows the far-field interpretation accuracy with respect to the time domain. In this experiment, 200 image frames from the test scene ③ are sampled consecutively at a frequency of 0.5 HZ. For each sampled frame, the accuracy is calculated as the number of correctly interpreted ground and object pixels divided by the number of all pixels in the far-field domain. The red curve shows the accuracy of a far-field model that uses the proposed on-line prototype (appearance + spatial context) building, and the blue curve corresponds to a far-field model that uses an offline prototype built from the initial frames of test scene ③.

It can be observed from Figure 21 that the accuracy of the off-line model deteriorates significantly from around 44 frames, while the accuracy of the on-line model remains relatively high throughout the experiment. The reason for the different behaviours in interpretation accuracy is due to the change in road appearance. In the first 40 frames, there is not much change in road appearance from the first few initial frames; therefore, the accuracy of the on-line and off-line model is similar. However, when there is significant change in road appearance from around frame 44, the off-line model fails to adapt to the changing environment, which leads to degradation in interpretation accuracy. In contrast, the on-line model achieves a steady accuracy above 90% despite the change in road appearance, showing its capability of adapting to the road environment.

**Figure 22:**
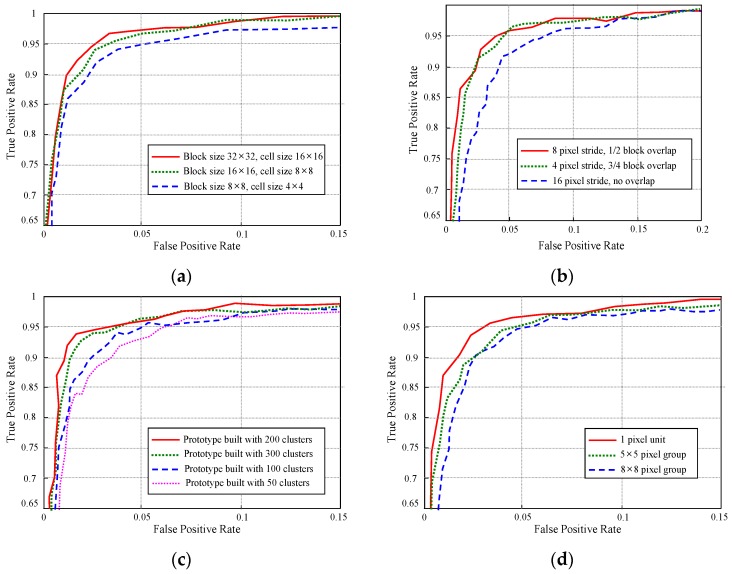
Far-field model performances with respect to the important model parameters. (**a**) Far-field model performances in different block sizes of the appearance prototype; (**b**) Far-field model performances in different matching strides; (**c**) Far-field model performances using different prototype sizes; (**d**) Far-field model performances in different block sizes of the spatial prototype.

Regarding the proposed far-field interpretation model, its interpretation performance is related to several important model parameters. Several experiments are conducted to determine the effects of these model parameters, and the results are shown in Figure 22.

The experiment result as shown in Figure 22a measures the far-field model performance with respect to the image block size, which is an important parameter that influences the interpretation performance of the appearance prototype. In this experiment, the sample images from test scene ② are used to run the far-field model with all other parameters fixed, only varying the image block size. It turns out that small block size as 8 × 8 is not large enough to contain distinct appearance features inside the block area. Therefore, the interpretation performance of 8 × 8 block is lower than that of 16 × 16 and 32 × 32. Although 32 × 32 block shows a little better performance compared with 16 × 16 block, 16×16 block size is adopted in our far-field model to achieve a balance between interpretation accuracy and interpretation resolution. Figure 22b shows an experiment on the stride length during the appearance prototype matching. This experiment is carried out using sample images from test scene ③. The image block size is set to 16 × 16 in the experiment. The experimental result demonstrates a very close performance for models that use 4-pixel and 8-pixel stride. Considering the moving efficiency during prototype matching, the 8-pixel stride length is used in the far-field model.

Another parameter that related to the far-field model performance is the size of the appearance prototype. The sample images from test scene ① are used to conduct this experiment on the prototype size. As shown in Figure 22c, if the prototype size is too small, it may not be able to represent the general appearance pattern comprehensively. Therefore, the model that uses a 50 cluster prototype behaves the worst in interpretation performance. However, if the prototype size is too large, the on-line update rate of some outdated clusters in the prototype will be decreased. This might increase the risk of making interpretation errors that are caused by matching with some outdated appearance patterns in the prototype. Based on the results of the experiment, a medium size of 150 clusters is generally used for the appearance prototype building. The performance of far-field model is also related to the size of pixel group that is used in building spatial context prototype. The experiment result in Figure 22d shows that the best performance is achieved by building spatial prototype in 1-pixel unit. And the neighbourhood histogram will deviate from the true spatial distribution when it is built using pixel groups. However, it takes a lot of computation cost for building and propagating spatial context in 1-pixel unit. Therefore, the 5 × 5 pixel group is used to achieve a balance between computation cost and interpretation performance.

**Figure 23:**
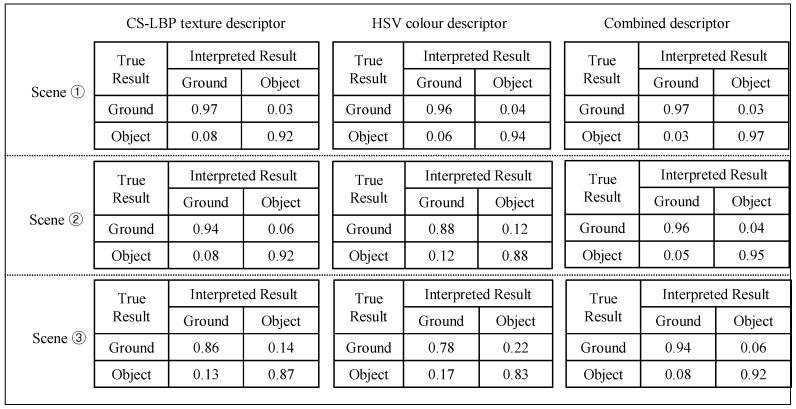
Far-field model performance using different appearance descriptors.

Finally, experiments are conducted on the interpretation performance regarding different types of descriptors. In one experiment, three types of appearance descriptors were applied to the sample images from the three test scenes; the average interpretation results are listed in Figure 23. In Figure 23, the first column shows the interpretation results obtained using only the CS-LBP descriptor, the second column shows the interpretation results of the colour histogram and the third column shows the interpretation results of the combined CS-LBP and colour histogram.

The interpretation results in Figure 23 show that the CS-LBP descriptor and colour histogram descriptor may compensate for each other in the appearance interpretation. For example, in test scene ①, the texture of ground and roadside objects (low wall on the left) is similar, while the colour is very different. Therefore, the CS-LBP descriptor behaves worse than the colour histogram descriptor. While in test scene ②, the opposite is true. Therefore, the combined descriptor can produce better results compared to a single type of descriptor alone.

**Figure 24:**
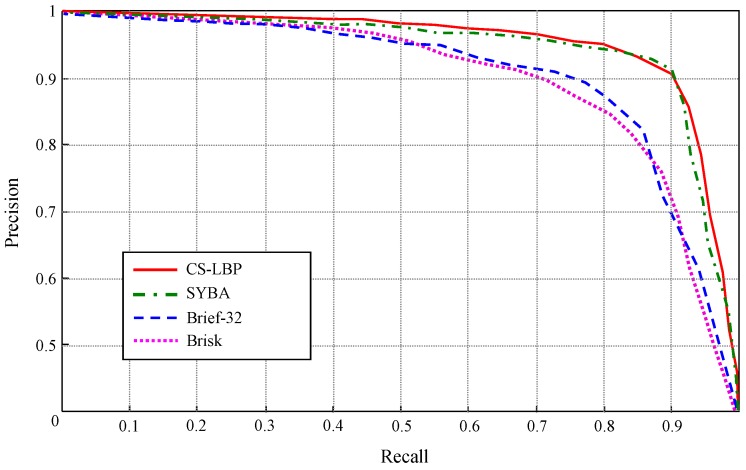
Far-field model performance using different binary descriptors.

In another experiment, the far-field interpretation performance using different types of binary descriptors are compared as shown in Figure 24. In this experiment, four types of binary descriptors are applied to the images in test scene ③, and the precision-recall curve of each binary descriptor is obtained by varying the decision threshold in a similar way that the ROC curves are obtained in Figure 20. When building the ground prototype, the feature points are detected on the top-view domain using FAST detector for all the binary descriptors, and all the binary descriptors are applied to a canonical image block size of 32 × 32.

As shown in Figure 24, CS-LBP and SYBA [28] descriptor achieve similar performance. At 90% recall, both of them maintain high precision level above 92%. While for Brief [29] and Brisk [30], their precision fall below 74% at 90% recall. Therefore, CS-LBP and SYBA show better discrimination capabilities for the test scenes. The reasons for this performance difference are mainly due to the descriptor structure. The CS-LBP descriptor captures gradient patterns across the diagonal direction in a neighbourhood, and the gradient patterns are represented in histograms concatenated from adjacent cells. Therefore, local textures with their spatial distributions can be well encoded in CS-LBP descriptor. Similarly, SYBA descriptor encodes local texture and their spatial distributions by using synthetic basis functions. In comparison, Brief descriptor takes the gray level difference at random pixel locations to build a binary string as the descriptor. Although it is easy to build and compare, the binary string built in this way loses some statistical and spatial properties of local textures. Therefore, the performance of Brief descriptor might be sufficient for key point matching, but for the task of texture representation and classification, the CS-LBP and SYBA descriptors turn out to be better choices.

### 5.2. Run-Time Performance of the Dual-Field Sensing Model

Significant effort is invested in implementing the model to make the dual-field sensing model really work for real guidance tasks. Many strategies and techniques are applied in the implementation to achieve a balance between the interpretation performance and the run-time performance. The near-field model proposed in [23] was re-implemented to allow sufficient run-time space for the far-field model computation.

**Figure 25:**
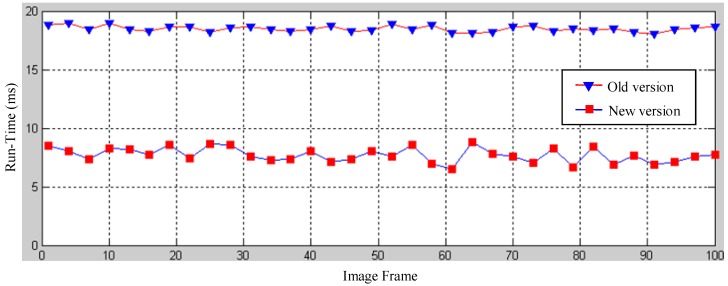
Run-time improvement of object profile separation in the near-field model.

One of the major improvements in the near-field model is the implementation of a scout-point-voting scheme for object profile separation. In the old version, a gradient-ascend maximisation of a smooth likelihood function was required to separate range data into smooth object profiles. In the old version, each range data point has to be moved to one of the local maxima of the smooth likelihood function using a gradient-ascend vector. Scout-point-voting is based on the fact that the number of local maxima is generally far less than the number of laser points, and most local maxima can be identified by moving only a few scout points. For a regular point, its convergence destination is voted using the destinations of scout-points that pass by its neighbourhood. Therefore, the majority of laser-points can be directly assigned to its local maxima without doing a gradient-ascent computation. A run-time comparison of the old and new versions of the object profile clustering is shown in Figure 25. It shows that the new version reduces the run-time to about half that of the old version using the scout-point voting scheme.

In the far-field model, the major implementation strategies used to optimise run-time performance are listed in Table 3. In addition, an integration of a multi-core programming technique and a graphical processing unit (GPU) programming technique was also used in the implementation to fully exploit the maximum capacity of the computing platform.

Finally, to evaluate the run time performance of the dual-field model, an optimised implementation is run on a laptop computer with the specifications shown in Table 4. The specification of the data frame is shown in Table 5, and sensor data collected from the test scene ② shown in Figure 19 are used to run this experiment. The model parameters are tuned to reach a balance between the interpretation accuracy and running time. The average run-time result for one data frame is listed in Table 6. It took about 197.93 ms for the dual-field model to process one data frame. This runtime performance achieves about 5 frames per second on average.

### 5.3. Guidance Performance of the Dual-Field Sensing Scheme

**Figure 26:**
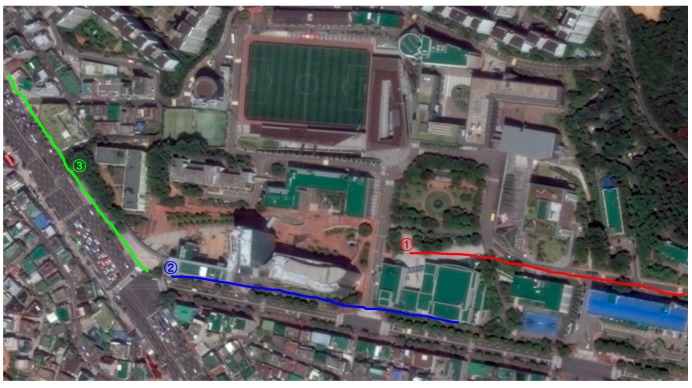
Selected paths for the field test around our campus.

To test the guidance performance of the dual-field sensing scheme, three urban paths around our campus are selected for the field test. These testing paths are shown in Figure 26, where path ① corresponds to test scene ① shown in Figure 19, and paths ② and ③ correspond to test scenes ② and ③, respectively.

Each test path spans a length of 250 m. The blind users who participated in the field test were unfamiliar with the test paths. During testing, each blind user was asked to carry the whole guidance system on their body, including the sensor platform and the notebook computer. Each blind user was supposed to walk along the test path by following the guidance messages provided by the system. To test the efficiency of the dual-field sensing scheme, each field test was run three times. In the first round, the blind users were asked to walk along the path using only a white cane. In the second round, the blind users conducted the test using our guidance system with only near-field sensing. In the third round, the blind users were required to use the guidance system with the dual-field sensing.

The field test was performed at around 11:00 a.m. in the morning and at 4:00 p.m. in the afternoon when there was a moderate number of moving objects, such as pedestrians on the road. There were also various static objects located along the testing paths. The time it took for the blind user to walk along each path was recorded, and the average time it took to walk along each path is shown in Figure 26.

**Figure 27:**
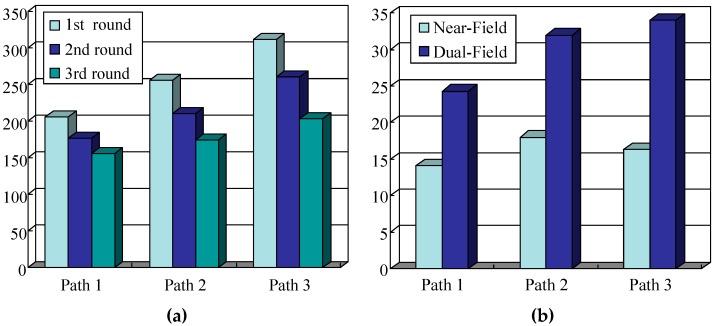
(**a**) Traveling time on each test path; (**b**) Increased efficiency for each path.

Figure 27a shows that the guidance system dramatically reduces the time it takes to walk along each path, and the more complex the road condition is, the more efficient the guidance system is. On path ①, where the road environment is simple, there is not much difference between using a white cane and the guidance system. However, on paths ② and path ③, where road conditions are more complex, the dual-field sensing scheme demonstrates its efficiency. The increased efficiency is measured as |*t*_g_-*t*_c_|/*t*_c_ quantitatively, where *t*_g_ is the traveling time when using the guidance system, and *t*_c_ is the traveling time when using a white cane. The efficiency evaluation result is shown in Figure 27b.

It can be observed that the dual-field sensing scheme provides extra efficiency compared to only near-field sensing. For example, on path ③, the near-field sensing module reduces the travelling time by 16.3%, and the dual-field sensing scheme reduces the travelling time by 34%. The field test results have proved the effectiveness of using the dual-field sensing scheme for guiding the blind people in an urban walking environment.

## 6. Conclusions

In this paper, a dual-field sensing scheme is proposed for a guidance system for blind people. It provides a practical solution to boost the scene interpretation capacity using very simple sensor configurations. In the proposed scheme, a 2D laser scanner and a camera are all the sensors that are needed to produce the dual-field interpretation of the scene. The near-field interpretation allows the white cane to be replaced by identifying ground and low-level objects in high resolution. The far-field interpretation increases the overall guidance efficiency by compensating the limited near-field interpretation scale. By using the dual-field sensing scheme, the guidance system can provide more advanced guidance to blind people. With the increased interpretation ability of the guidance system, more efficient user feedback is required to convey the interpreted environmental information. Therefore, our future work will be devoted to further improving the user feedback scheme, with the goal of making user feedback perception as natural as possible.

## Figures and Tables

**Table 1:** Guidance message generation scheme.

Message Type	Message Content		Delivery Modality	Delivery Timing	Message Prototype
Essential	Nearest Object Location		Tactile/Acoustic-Beeper	Continuous Real-Time	The frequency of the acoustic beeper/tactile units encodes distance, and the location of beeper signal in the acoustic space encodes orientation./The location of tactile units in the tactile array encodes orientation.
	Safe Direction		Verbal/Stereo Sound	Event-Triggered	‘’Go (1 o’clock).’’/Stereo source location in the acoustic space generated between two earphones.
User Context	Alert Message		Verbal	Event-Triggered	“Large departure attention!”/“Collision attention!”
Road Context	*ψ*	General Condition	Verbal	Periodic	“The road condition is (easy/ordinary/tough).”
	*d*	Road Width	Verbal	Periodic	“The road ahead is (narrow/wide/very wide).”
	*θ*	Road Orientation	Verbal	Periodic	“The road ahead turns (11 o’clock).”
	*ω*	Crowdedness	Verbal	Periodic	“The road ahead is (spacious/crowded/highly crowded).”
	*η*	Dominant Object Type	Verbal	Periodic	“The road ahead is dominant by (vertical object/lateral object/person).”

**Table 2:** Saliency of context messages in different road contexts.

Easy Context			Ordinary Context			Tough Context		
S1	S2	S3	S1	S2	S3	S1	S2	S3
*ω*.wide	*ω*.normal	*ω*.narrow	*ω*.normal	*ω*.narrow	*ω*.wide	*ω*.narrow	*ω*.normal	*ω*.wide
*ρ.*low	*ρ.*medium	*ρ.*high	*ρ.*medium	*ρ.*high	*ρ.*low	*ρ.*high	*ρ.*medium	*ρ.*low
*θ.*small	*θ.*medium	*θ.*high	*θ.*medium	*θ.*high	*θ.*small	*θ.*high	*θ.*medium	*θ.*small
*η*.low	*η*.medium	*η*.high	*η*.medium	*η*.high	*η*.low	*η*.high	*η*.medium	*η*.low

**Table 3:** Some implementation strategies in the far-field model.

Major Modules	Implementation Strategy
Top-view mapping	Pre-configured mapping table.
On-line prototype updating	On-line prototype update is triggered only at some key frames.
Appearance Prototype matching	Approximate nearest neighbor search based on the randomized k-d forest or k-means tree.
Spatial context propagation	Apply spatial propagation with 5 × 5 pixel group as a unit.

**Table 4:** Platform specification of the run-time experiment.

Platform Component	Specifications
CPU (Central Processing Unit)	Intel core i7@ 3.5GHZ
RAM (Random Access Memory)	16GB DDR4
GPU (Graphic Processing Unit)	Nividia Quadro M1000M
OS (Operating System)	Windows 7 64bit
C++ Compiler	Microsoft VC++ 2013

**Table 5:** Data frame specification.

Data Frame Component	Specifications
Laser range data	480 range data points
Original-view image data	640 × 480 HSV color space
Top-view image data	370 × 210 HSV color space

**Table 6:** Average run time performance of the dual-field sensing model.

Model Component	Module	Function	Runtime/Function	Runtime /Module
Near-field interpretation	Ground estimation	Ground model fitting	8.74 ms	8.74 ms
	Object detection	Object range data labeling	6.82 ms	17.39 ms
		Object profile separation	10.57 ms	
	Object classification	Multimodal profile histogram	10.67 ms	41.98 ms
		Generic object classification	8.46 ms	
		Pedestrian classification	22.85 ms	
	Near-field total	All near-field functions	68.11 ms	68.11 ms
Far-field interpretation	Inverse Perspective Transform	Top-view mapping	10.47 ms	10.47 ms
	Cross-field scene interpretation	Appearance prototype matching	56.34 ms	91.58 ms
		Spatial prototype propagation	35.24 ms	
	On-line prototype updating	Appearance prototype updating	21.42 ms	38.24 ms
		Spatial prototype updating	16.82 ms	
	Far-field total	All far-field functions	129.82 ms	129.82 ms
Dual-field interpretation	All dual-field modules	All dual-field model functions	197.93 ms	197.93 ms
